# Does higher education make women sicker? A study of the gender gap in sickness absence within educational groups

**DOI:** 10.1371/journal.pone.0303852

**Published:** 2024-06-10

**Authors:** Charlotta Magnusson, Roujman Shahbazian, Sara Kjellsson

**Affiliations:** 1 Institute for Social Research (SOFI), Stockholm University, Stockholm, Sweden; 2 Department of Sociology, Faculty of Social Science, Uppsala University, Uppsala, Sweden; 3 Department of Sociology, Faculty of Social Science, University of Munich (LMU), Munich, Germany; University of Oxford, UNITED KINGDOM

## Abstract

This study describes changes in the withdrawal of sickness benefits among men and women in Sweden over a period of three decades (1994–2018), based on administrative data. During this period there was a gender gap in the takeout of sickness benefits to women’s disadvantages in all age groups as well as educational groups. The gap was particularly large between men and women with secondary education in the ages 30 to 39. The general gender gap in sickness absence is larger today compared to 1994. The development, after 2010, was mainly driven by a larger increase in sick leave among women with secondary education, both in relation to men with secondary education and in relation to women with both lower and higher levels of education. For women with secondary education, sick leave does not seem to vary according to age. Thus, in this educational group, women of child-rearing age are not more prone to take sick leave than other age groups.

## Introduction

Sick leave and its consequences are a well-established area in health research, with significant implications for individuals, workplaces, and society as a whole. Previous studies have established links between sickness absence and various factors such as individual career progression, work performance, and financial well-being [1, 2], as well as, increased working cost and decreased productivity [3, 4]. From a societal perspective, sickness absence can also result in higher healthcare costs and decreased economic growth.

A notable aspect within this research domain is the gender disparities in sick leave rates, with women having higher rates than men [5, 6]. This gender gap has been observed to increase since the 1980s [7], particularly in countries with high female labor market participation [8]. We also know that the gender gap in sickness benefits accelerates, or comes along, with the birth of the first child [9]. That sickness absence is related to education is also clear as studies point out an educational gradient in sick leave for both men and women, where low educated tend to have a higher risk of sickness absence [10]. Despite this extensive research, there are limited findings on how the gender gap in sick leave has evolved over time and across different educational and age groups.

To fill this gap, we use national administrative data to explore gender differences in sick leave in Sweden over three decades, across educational and age groups. Focusing on Sweden is crucial for several reasons. First, in Sweden women take twice as many sick days as men [11]. Second, Sweden stands out from most other countries because of its high level of gender equality [12]. Both women’s participation in paid work and higher education took place earlier in Sweden compared to many other countries. Today a majority of Swedish women hold upper secondary education, and have surpassed men’s educational levels [13]. Therefore, it is vital to examine the gender gap in sick leave across educational groups. Third, Sweden has had consistent and nearly equal labor market participation rates for both genders during the study period, 1994–2018 [14]. Currently, 85 percent of Swedish women are in the workforce, indicating a long-standing high employment rate. Fourth, while the socio-economic status health gradient traditionally relies on occupational-based measures, recent findings from Sweden challenge this approach. Contrary to the conventional assumption that individuals settle into a stable occupation or class position after the age of 35, the findings of Bihagen and colleagues [15] indicate more fluctuation than assumed. Using educational attainment as a measure of socio-economic status circumvents this issue, providing a more consistent and stable metric.

## Background

During the past decades, there has been an educational expansion in the labor market in most societies [16], and in Sweden, this is certainly true. From the 1990s, the share of men and women with at least three years of postsecondary education has increased from 11 percent to 32 percent. This increase has been particularly large among women. In 1990, 11 percent of all women belonged to this group and by 2020 this number had increased to 34 percent. For men, the increase has gone from 11 to 22 percent [17].

High education is, in general, related to better living conditions and well-being [18–20]. Levels of health, both subjectively and objectively measured, generally rise with increasing levels of education [19, 20] and this relationship can be understood through a variety of mechanisms [21]. For instance, abilities achieved through education, such as learned effectiveness, can be drawn upon to further health and are thus linked to lower risks of ill health [20]. Higher educated also tend to display a stronger sense of control and few health-negative behaviors, such as smoking, compared to the lower educated [21].

Different levels of education, furthermore, grant different opportunities in the labor market. Typically, jobs with low formal demands for educational qualifications tend to be occupations that involve a larger degree of demanding work conditions, which partly mediates the relationship between education and health [22]. The relationship between occupation and health is, in itself, multifaceted but a large part is connected to variations in working conditions, including both physical and psychosocial environments and demands, as well as levels of income [20, 23, 24]. In the Swedish context, we can note an improvement in physically demanding working conditions during the latter part of the 20^th^ century, while the same cannot be said for psychosocial demand. The most common reasons for sick leave in Sweden are, also, due to psychiatric diagnoses [25, 26]. Thus, mental health problems represent the most frequent cause of sick leave for women and the second most frequent cause for men [27].

Accounts of differences in working conditions are, generally, not reported by educational level but by occupational or class position. The same can be said for differences in sickness absence, which is mostly researched in relation to occupation or working conditions. For educational differences in sick leave from work, the occupational positions for men and women with different educational levels can be of particular interest. In what occupations do we find men and women with different educational levels, and what types of working conditions do they have? In the Swedish labor market, women in all educational groups typically work within the care, healthcare, or educational sectors but at different levels of skill and seniority, from child-minders and nurse’s aides to teachers, nurses, and health professionals. Men with compulsory education are found primarily in transport, construction, and retail; secondary educated men work in engineering, business, and construction, while the highest educated group are computing or business professionals, architects, and civil engineers [28]. See Table A1 in the S1 Appendix for a description of the three most common occupations in our data within each education category.

According to the clear health gradient in relation to education and together with the educational expansion, it is likely to assume a decrease in sick leave over time–especially among women. There is however no indication of such a change. Mastekaasa [6] investigated gender differences in sickness absence over time, in eight European countries (Belgium, France, Ireland, Italy, the Netherlands, Portugal, Spain, and the UK), and found that the gender gap in sickness absence, to women’s disadvantages, had increased in five of them.

Angelov and colleagues [7] ascribe a large part of the gender gap in sickness absence to gender differences in responsibility for children and showed that mothers increase their sickness absence more than fathers after the first child is born. Despite the fact that women have approached men’s time in paid work and have a higher educational level women still take greater responsibility for children and unpaid household work [29]. However, previous research has shown housework to be more equally divided in couples where both partners have higher education [30]. Highly educated women tend to do less household work [31] and have a stronger connection to paid labor. Thus, it is likely to assume sick leave is most present among women with less education both according to the educational gradient in health and according to their weaker connection to paid labor and larger responsibility for unpaid work.

However, as women still, in general, have the main responsibilities for unpaid work their improvement in educational level and stronger integration into the paid labor force might increase the total workload for all women regardless of education. The total workload could also be more intense among highly educated as part-time work is less common in this group. The structural changes during recent decades may have produced a situation in which women generally have come to experience both a deterioration in their working conditions and an intensification in their total workload, thus increasing the risk for ill-health [32]. From this perspective, it is unclear whether or not higher education would result in better health, and, by extension, a lower level of sick leave level among women.

This article is descriptive, displaying trends during the mid-1990s until today in the gender difference in sickness absence within age and educational groups. This is highly relevant due to the structural change that has taken place during this time period, where the share of women in higher education has increased dramatically. Unlike prior studies we not only look at the gender gap in sickness absence for different educational groups, we also consider variations in sickness absence among men and women separately. This is important, as it is likely to assume the development of sick leave to vary not only between genders but also within genders depending on education.

## Data and methods

The study uses national-wide administrative data from the Longitudinal Integrated Databases for Health Insurance and Labor Market Studies (LISA) from 1994 to 2018, provided by Statistics Sweden. We gather data on individuals’ sex, age, education, and annual sick days. Our focus is on individuals aged 20–59, living in Sweden during 1994 to 2018 and eligible for sickness benefits. We began in 1994 due to data availability and concluded in 2018 to avoid the Covid-19 pandemic’s impact on sick leave. Thus, the study’s findings aren’t influenced by the pandemic.

### Variables

#### Sickness allowance

In Sweden, individuals with income from work, unemployment, or parental leave can receive sickness benefits if illness prevents them from working. The first day of absence from work due to illness is a quarantine day without benefits, and the next 13 days are paid by the employer. From 14^th^ day onward, the Swedish Social Insurance Agency provides benefits given a physician’s certificate. We only use information about sick leave that has been provided by the Social Insurance Agency, i.e., only those who have been absent from work due to illness 14 days or more are counted as had been on sick leave. The benefit covers around 80% of one’s income (up to a certain limit) and can for part- or full-time working hours (i.e., 25, 50, 75, or 100%). We analyze “net days” of sickness absence. However, analyses based on “gross days” yield similar results.

#### Education

Level of education is divided into three groups, pre-secondary education (low educated, Edu 1), secondary education (intermediate educated, Edu 2), and postsecondary education (highly educated, Edu 3).

#### Age

When we use categories of age, we divide individuals into four age groups: age 20–29, age 30–39, age 40–49, and age 50–59.

### Method

From 1994 to 2018, we compute the annual percentages of men and women receiving at least one day of sickness benefits. These calculations were also divided by gender, age, and educational group. By calculating the percentage that received sickness benefits for each year, we eluded the problem of potential changes in sickness benefits over time. Furthermore, as we are interested in how the gender gap in sickness absence has evolved and not the absolute number of individuals on sick leave, changes in the insurance system are of minor importance. For the defined groups (sex, age, and education level) we calculated the annual gender gap in sick leave by subtracting the percentage of men (by age and/or education) from the corresponding women percentage.

In the presentation of our results, we first illustrate the general gender gap in sick leave over time, divided by age. Thereafter, we focus on the level of education, where we show both the gender gap in sick leave and the percentage of men and women, by educational level, which had any take-out of sick leave. In the third step, we combine both age groups and levels of education, and these patterns are displayed for the gender gap as well as for men and women, respectively.

## Results

**Fig 1:**
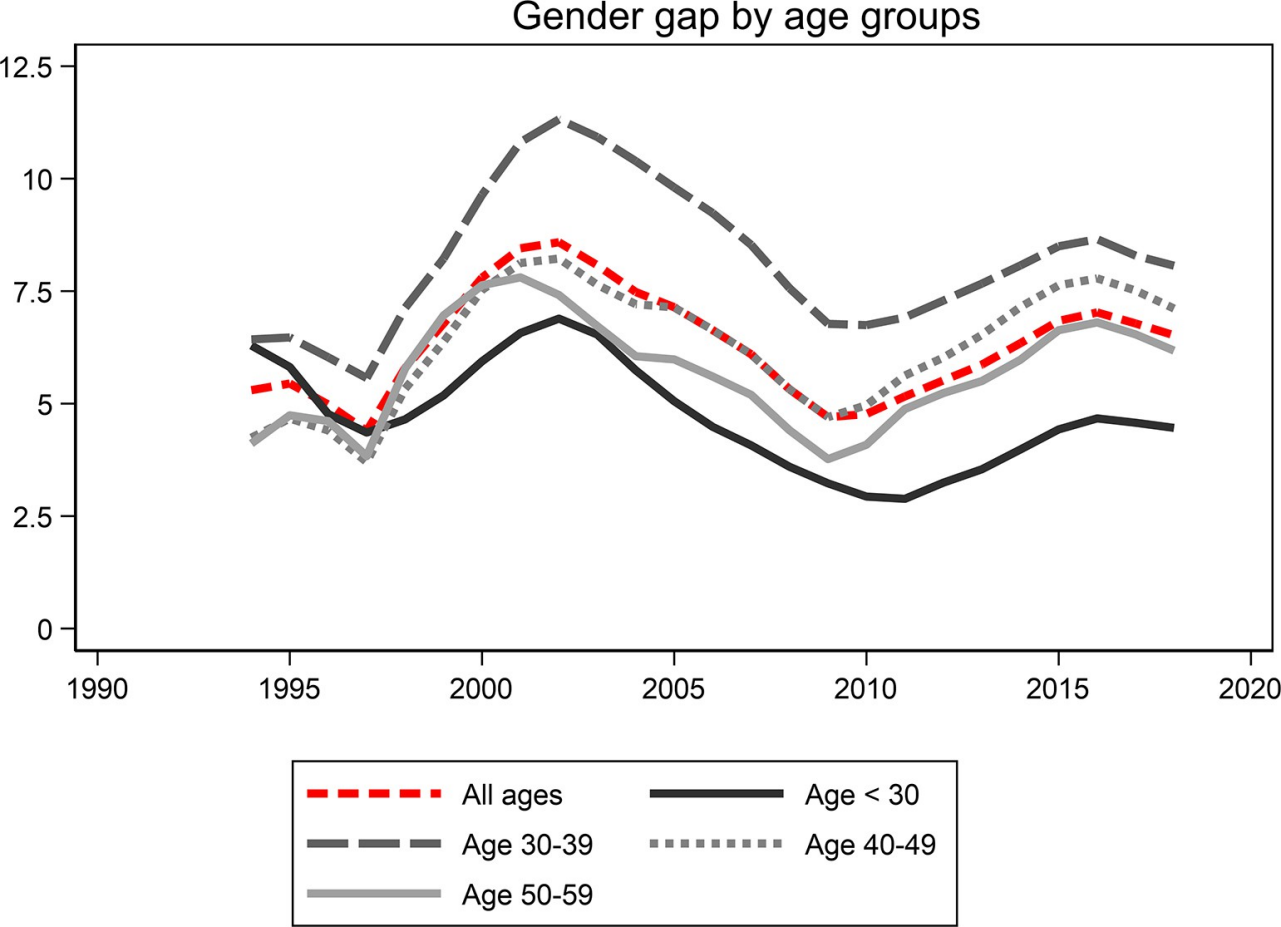
Gender gap in sick leave absence by age groups. The gender gap in sick leave (women–men) is divided into four age groups: below age 30, 30–39, 40–49, and 50–59. The red line includes all ages. The y-axis is percent (0–12.5%), and the x-axis is years.

Fig 1 shows a gender gap in sickness absence, with women taking out more sickness benefits, from around five percent in 1994 to six percent 2018. The gender gap is prevalent across all age groups, but is more pronounced among those aged 30 to 39. The gender gap peaks in the early 2000s and again around 2015, consistent across age groups.

**Fig 2:**
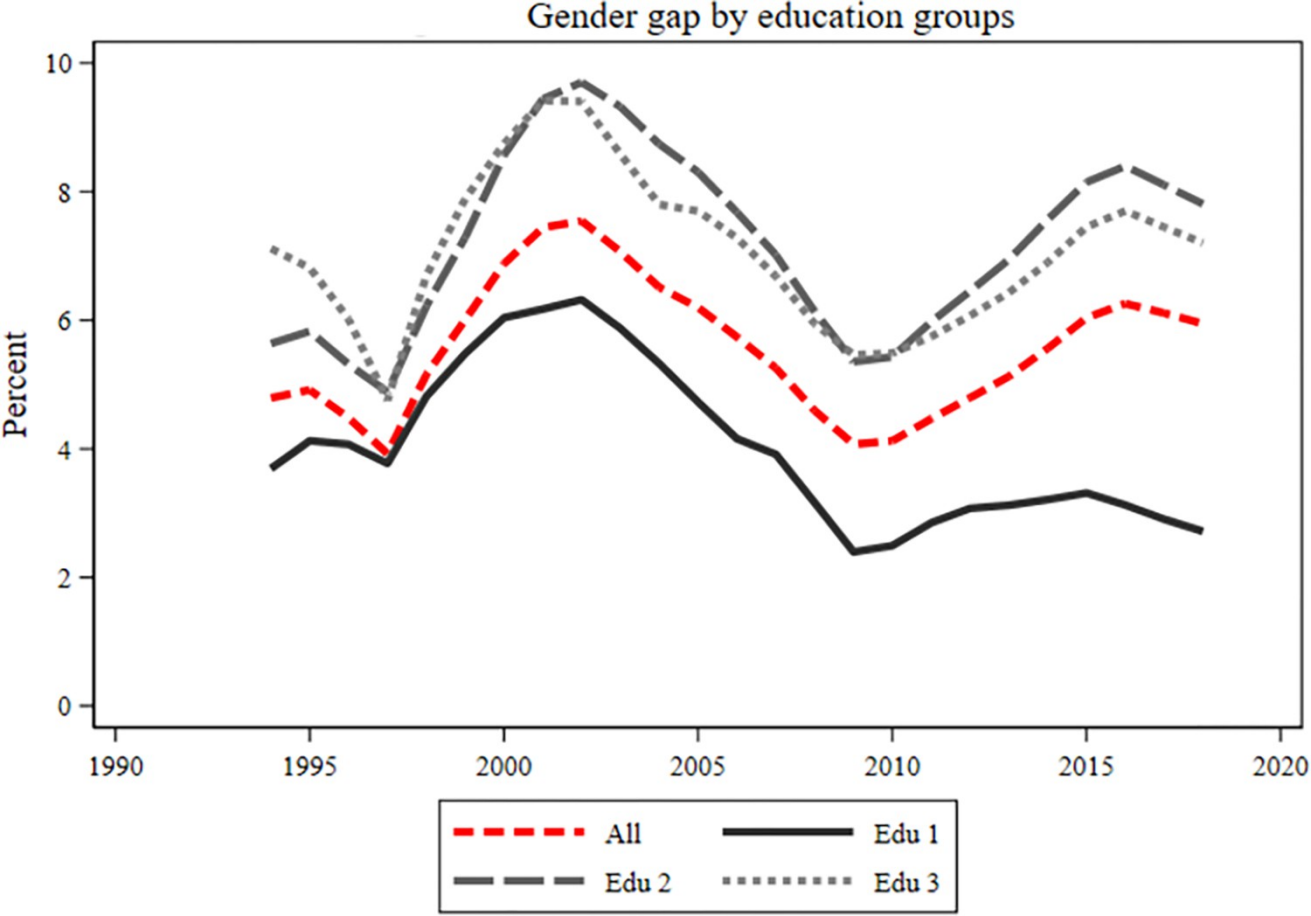
Gender gap in sick leave absence by educational groups. Fig 2 shows the gender gap in sick leave (women–men) divided by education groups. The three education groups are low educated (edu 1), intermediate educated (edu 2), and highly educated (edu 3). The red line includes all education groups. On the y-axis is percent and on the x-axis is years.

We next examine sickness benefits by educational groups. Fig 2 shows a gender gap to women’s disadvantages in all educational groups. However, the gender gap is smallest among low-educated, especially in recent years, around 3 percent after 2010. Both secondary and postsecondary educational groups display similar gender gaps over time, though recent trends suggest a widening gap for those with a postsecondary education level. For this group, 2018 levels (8 percent) are higher than 1994 (5,5 percent). Thus, women with post-secondary education had a larger take-out of sickness benefits compared to men with post-secondary level of education.

**Fig 3:**
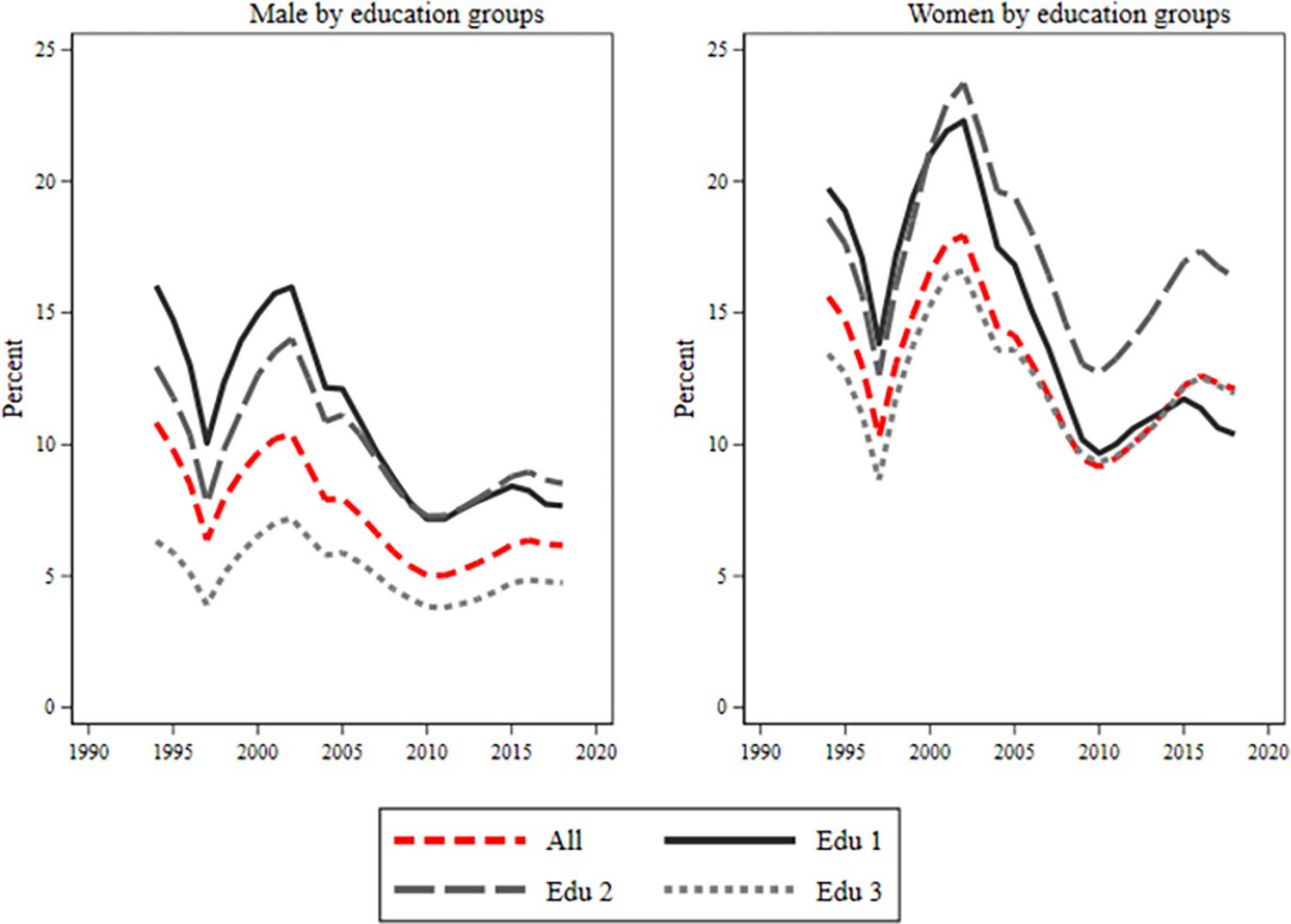
Men and women’s in sick leave absence by educational groups. Fig 3 shows sick leave (separated by gender) by education groups. The three education groups are low educated (edu 1), intermediate educated (edu 2), and highly educated (edu 3). The red line includes all education groups. On the y-axis is percent and on the x-axis is years.

The separate graphs for women and men (Fig 3) initially show a social gradient in sickness absence for both men and women based on educational level. Those with pre-secondary education have the highest sickness absence, while those with the highest education have the lowest. The sickness absence had a peak at the beginning of the early 2000s in all educational levels and for both genders; all men had around 10 percent while women had around 18 percent. Yet, around 2015, women, particularly those with secondary education, experienced another peak. The gap between low-educated women and those with postsecondary education narrowed post-2000, with the former group having fewer sickness absences by the end of the period. During the study period, low-educated women’s use of sick days decreased. For this group, 2018 levels (10 percent) are lower than 1994 (20 percent). For those with post-secondary education, 2018’s takeout mirrored 1994’s. Since the year 2000, women with upper secondary education have had the highest sickness benefit withdrawals.

Among men (Fig 3), highly educated people clearly had the lowest proportion of sick leave during the entire period, fluctuating between 4 to 6 percent. At the beginning of the period until 2007 the largest take-out of sickness benefits was among men with the lowest level of education, fluctuating between 10–16 percent. Since 2007–2008 the difference between those with pre-secondary education and secondary education has been negligible, but in recent years the proportion of sick leave was slightly larger among men with secondary education.

**Fig 4:**
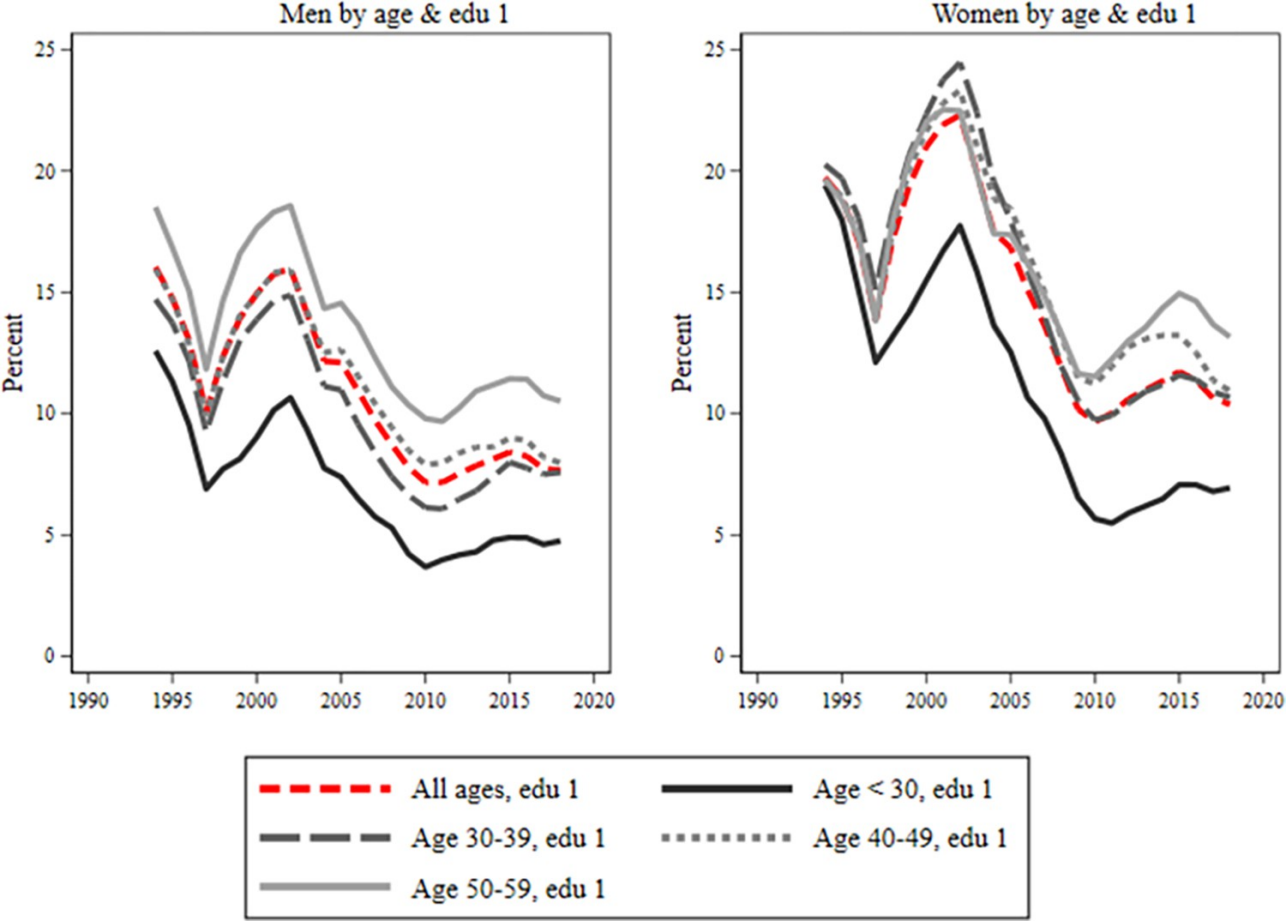
Sick leave for men and women with pre-secondary education by age groups. Each figure is divided into four age groups: below age 30, 30–39, 40–49, 50–59. The red line includes all ages. On the y-axis is percent (0–25%) and on the x-axis is years.

**Fig 5:**
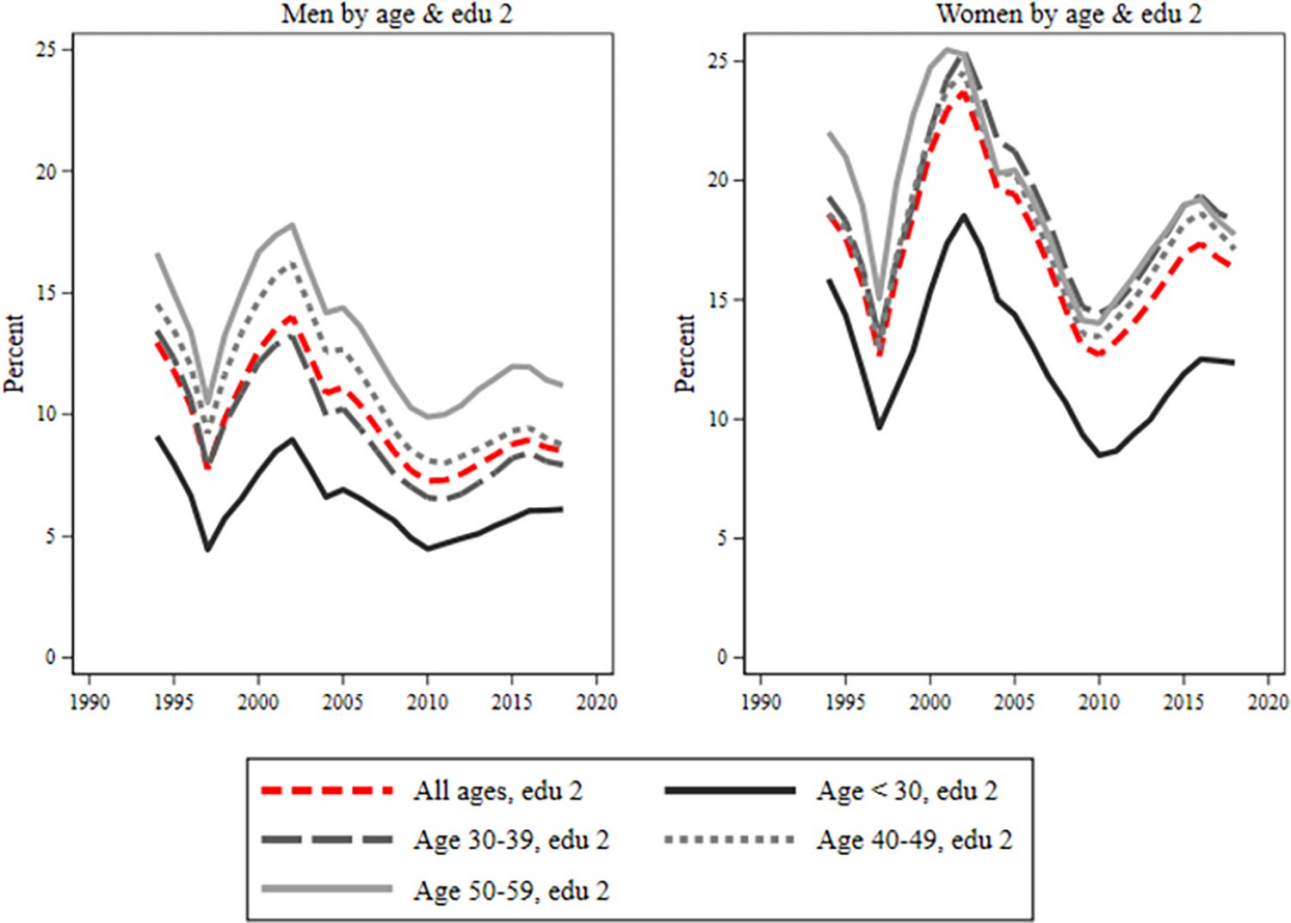
Sick leave for men and women with secondary education by age groups. Each figure is divided into four age groups: below age 30, 30–39, 40–49, 50–59. The red line includes all ages. On the y-axis is percent (0–25%) and on the x-axis is years.

**Fig 6:**
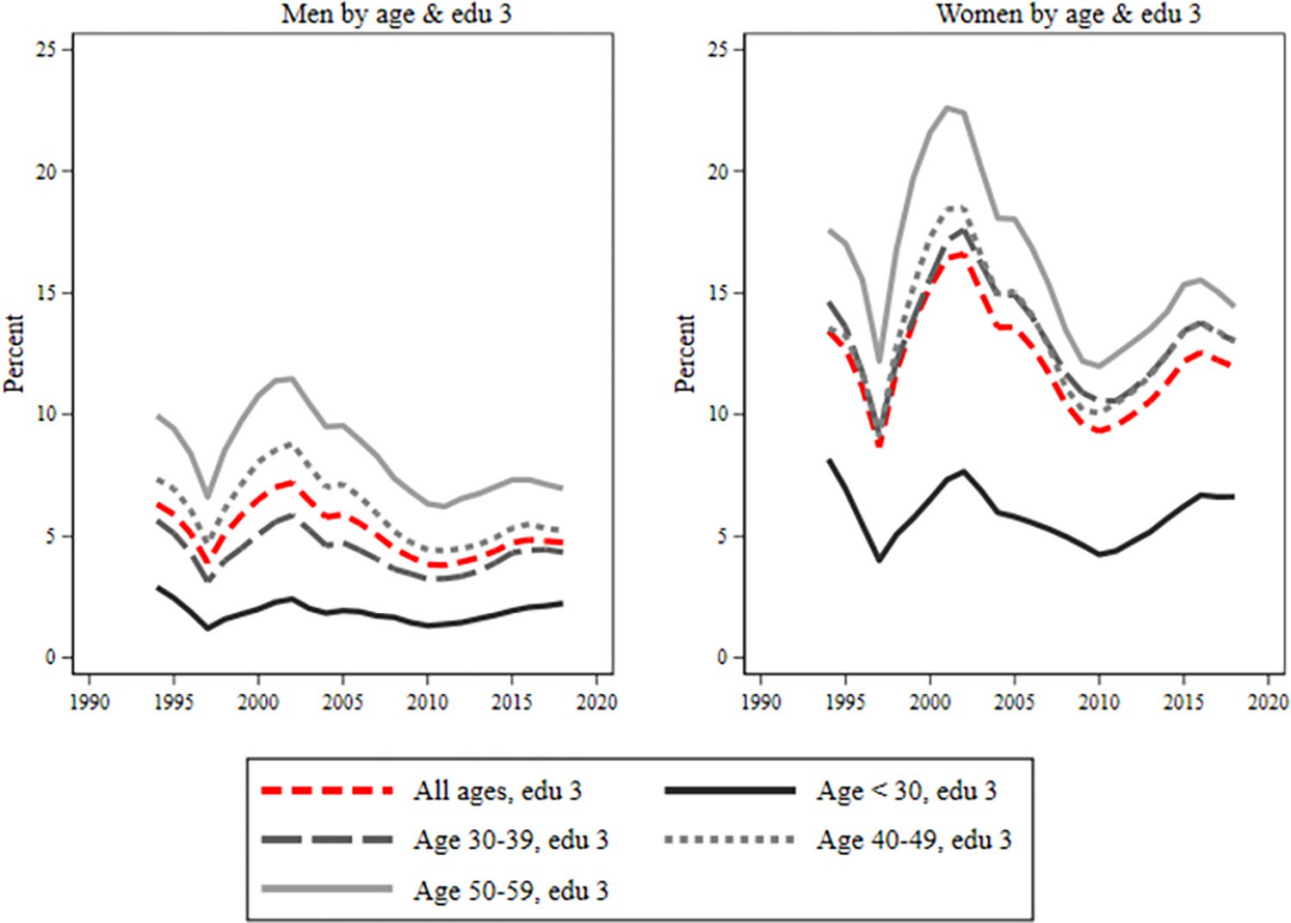
Sick leave for men and women with post-secondary education by age groups. Each figure is divided into four age groups: below age 30, 30–39, 40–49, 50–59. The red line includes all ages. On the y-axis is percent (0–25%) and on the x-axis is years.

During the early 2000s, both men and women experienced a reduction in sick leave across all age and educational groups. However, post-2010, women’s sickness absence increases (Figs 4–6). Young individuals consistently had the lowest sick leave rates, regardless of educational level. Low-educated women, Fig 4, showed minimal age groups differences until 2008–2009. After that, there was a tendency for a larger take-out among the oldest group of women. Women with secondary education, Fig 5, displayed similar rates across age groups, except younger ones had a slightly lower rate. Highly educated women, Fig 6. had a noticeable age gradient, but 30–39 and 40–49 age groups were similar. Conversely, among men, there was a classic age gradient in sick leave across educational groups where younger men had the lowest, and older men had the highest sick leave rates.

Overall, there was a gender gap in sickness absence for all ages and in all educational groups. However, there was some variation in the patterns displayed over time, particularly between those with pre-secondary education and those with secondary or post-secondary education. For those with pre-secondary education, the results showed a large gender difference from the mid-1990s in the younger ages. However, this difference among the young converged during the early 2000s. Since the beginning of the 2000s, the gender gap has been largest in the 30 to 39 age group, both among the highly educated and among those with secondary education. Compared with the low educated was the trend among those educational groups almost the opposite, displaying more convergence at the start of the period and with larger differences between age groups at the end of the period. Our results furthermore showed a particularly large gender gap between men and women with secondary education in the ages of 30 to 39. The gender gap in this group was about 12 percent around 2000, and, after a short decline around 2010, it was almost as high year 2015.

## Discussion

This study outlines the trends in sickness allowance in Sweden for men and women by age and educational groups from 1994 to 2018. The most significant finding is that the gender gap in sickness absence in Sweden has become more pronounced. This development is mainly due to an increase in sickness absence after 2010 among women with upper secondary education, both compared to their male counterparts and to women with different levels of education.

The largest gender gap was observed in the 30–39 age group. However, the patterns become less apparent when considering age and education groups separately. The key takeaway is that the link between age and sick leave varies according to the level of education. Women with secondary education have the largest rates of sick leave, yet within this group, age does not drastically affect the rate. Moreover, post-2010, women with secondary education experienced a significant rise in sick leave, with minimal age-related difference for those over 30.

While, we cannot discern causal mechanisms behind these patterns, it is plausible that the increased absence among these women relates to work conditions in jobs typically held after secondary education, since education often dictates occupational paths. Prior research indicates that physically demanding work conditions, common in male-dominated fields, have improved. However, psychosocial demanding working conditions, prevalent in female-dominated occupations, have not seen similar improvements. In general, women more often than men report experiencing high demands and limited resources in their work life [33]. A study comparing work conditions from 1974 and 2010 [34] reveals increasing “negative stress”–i.e., high mental demands in combination with restricted opportunities to make decisions at work–for both women and men, but this negative stress is more pronounced for women. Whether conditions for women with secondary education have deteriorated remains unanswered and warrants further investigation. The support for poorer working conditions among women is not unequivocal. For instance, Mastekaasa and Olsen [35] found no support for that the gender gap in sick leave in Finland is due to women’s work environment. Laaksonen and colleagues [36] noted that working conditions influenced short-term sick leaves more than long-term absences.

The study highlights the largest difference in sickness absence between men and women aged 30–39, often a family-starting age. This aligns with Angelov and colleagues [7], emphasizing child-rearing’s impact on women’s sick leave. Yet, the trend among women varies with age and educational level. For women with secondary education, age differences are minimal throughout the whole observation period. Thus, women of child-rearing age in this group are not more prone to take sick leave than other age groups. This hints that attributing the gender gap in sick leave primarily to parenting is a less fitting explanation, especially for this educational group. Additionally, older women with either pre- or post-secondary education take more sick leave, an age group where child-rearing is unlikely the cause.

The increased sick leave among women may be due to selection. As Pavalko and colleagues [37] demonstrated using U.S. data, the rise in health problems among employed women might stem from previously non-employed women with health concerns joining the workforce. However, one advantage here is that we consider a period when women were already established in the labor market. By 1994, the studies onset, 71 percent of Swedish women were employed versus 73 percent of men, and these rates grew by 2018 to 77 percent for women and 80 percent for men [14]. Given that the significant surge in women’s employment predates the study period, most adverse health-based selection likely occurred before the period. Yet, selection might still affect the results, especially for the oldest age group.

Another factor is reversed causality: the less educated may have more pre-existing health issues [38]. Determining the significance of each causal direction is challenging [39, 40], but both might operate simultaneously [41]. Notable, by the end of the study period, women with lower education took fewer sick leaves than their highly educated counterparts. Moreover, the Swedish social insurance system’s design likely affects the share of withdrawals [42, 43], where sick leave tends to increase with higher compensation levels [43]. However, the relationship is not unequivocal. Lindwall and Marklund (2011) studied the number of sick leaves during the period 1992–2008 and found that although specific changes in the generosity of the sickness insurance system were important in some cases, there were no consistent relationships between the generosity of the sickness insurance system and the number of sick days. Nor is there clear evidence that possible reforms affect men and women differently [44]. Though, there is some support for men being more sensitive to changes in remuneration [43]. However, the design of the social insurance system can affect differences in withdrawals between educational groups. As the less educated are often low-income earners, they may be more sensitive to loss of income. This could be one reason why the use of sick-days among low-educated is lower compared to highly-educated women towards the end of the study period.

A shortcoming with these analyses is that we only point out trends and cannot discern the mechanisms behind them. However, the patterns found here indicated that the gap in sickness absence both within and between genders differs according to age and educational level and that we need to consider the interplay of gender, age, and educational level to get a deeper understanding of gender differences in sick leave. Thus, sickness absence seems not to have a clear gradient where higher education clearly entails a reduced risk of sick leave, especially not so for women.

## Supporting information

S1 Checklist Human participants research checklist. (DOCX)

S1 Appendix (DOCX)
