# Supplementary material for: Does higher education make women sicker? A study of the gender gap in sickness absence within educational groups
Source: PLoS One. 2024 Jun 10;19(6):e0303852. doi: 10.1371/journal.pone.0303852 (PMC11164392; doi:10.1371/journal.pone.0303852)
Supplement: S1 Appendix — (DOCX) [file pone.0303852.s002.docx]

Appendix

| Table A1: The three most common occupations, by gender and level of education | | | | | | | |
| --- | --- | --- | --- | --- | --- | --- | --- |
|  |  |  |  |  |  |  |  |
|  |  | **Year 2001** | | **Year 2009** | | **Year 2018** | |
|  |  | **Men** | **Women** | **Men** | **Women** | **Men** | **Women** |
|  |  |  |  |  |  |  |  |
| **Edu 1** | **No 1** | Motor-vehicle drivers (832) | Personal care & related workers (513) | Motor-vehicle drivers (832) | Personal care & related workers (513) | Carpenter, bricklayer & construction worker  (711) | Cleaners & home service staff, etc. (911) |
|  | **No 2** | Building finishers & related trades workers (712) | Shop & stall salespersons & demonstrators (522) | Building finishers & related trades workers (712) | Helpers & cleaners (912) | Warehouse staff & transport managers, etc.  (432) | Store staff (522) |
|  | **No 3** | Building finishers & related trades workers (713) | Helpers & cleaners (912) | Building finishers & related trades workers (713) | Shop & stall salespersons & demonstrators (522) | Truck & bus driver  (833) | Nursing assistants (533) |
|  |  |  |  |  |  |  |  |
| **Edu 2** | **No 1** | Physical & engineering science technicians (311) | Personal care & related workers (513) | Building finishers & related trades workers (712) | Personal care & related workers (513) | Ceiling fitters, floor installers & plumbing fitters etc. (712) | Assistant nurses  (532) |
|  | **No 2** | Finance & sales associate professionals (341) | Shop & stall salespersons & demonstrators (522) | Building finishers & related trades workers (713) | Shop & stall salespersons & demonstrators (522) | Painters, painters, etc. (713) | Store staff (522) |
|  | **No 3** | Building finishers & related trades workers (713) | Other office clerks (419) | Finance & sales associate professionals (341) | Other office clerks (419) | Treatment assistants & pastors (341) | Office assistants & secretaries (411) |
|  |  |  |  |  |  |  |  |
| **Edu 3** | **No 1** | Physical & engineering science technicians (311) | Primary education teaching professionals (233) | Computing professionals (213) | Pre-primary education teaching associate professionals (331) | IT architects, system developers &  test leader etc. (251) | Primary education teaching (234) |
|  | **No 2** | Computing professionals (213) | Pre-primary education teaching associate professionals (331) | Architects, engineers & related professionals (214) | Primary education teaching professionals (233) | Civil-Engineers (214) | Nursing associate professionals (222) |
|  | **No 3** | Architects, engineers & related professionals (214) | Nursing associate professionals (323) | Physical & engineering science technicians (311) | Nursing associate professionals (323) | Engineers & technicians  (311) | Organizational developers, HR specialists etc. (242) |
|  |  |  |  |  |  |  |  |
| Note: The table shows the three most common occupations in the study within each level of education, separately for men and women. For the years 2001 and 2009 *the Swedish Standard* *Classification of Occupations 1996* (SSYK-96) is used. For 2018, the *Swedish Standard Classification of Occupations 2012* (SSYK-2012) is used. | | | | | | | |
